# Sleep Medication Use and Associated Factors Among Adults with Insomnia Symptoms: A Cross-Sectional Study in Brazil

**DOI:** 10.1055/s-0046-1825529

**Published:** 2026-07-24

**Authors:** Ana Flávia Bonini, Renatha El Rafihi-Ferreira, Helder Sérgio Lira Soares Filho, Andrea C. Toscanini, Rosa Hasan

**Affiliations:** 1 Psychosocial Care Center (CAPS), Secretaria Municipal da Saúde, São Paulo, SP, Brazil; 2 Department of Clinical Psychology, Universidade de São Paulo (USP), São Paulo, SP, Brazil; 3 Department of Psychiatry, Universidade deSão Paulo (USP), São Paulo, SP, Brazil; 4 Sleep Clinic, Instituto de Psiquiatria, Hospital das Clínicas, Faculdade de Medicina, Universidade de São Paulo (USP), São Paulo, SP, Brazil

**Keywords:** insomnia, hypnotics, behavioral therapy, medication use, sleep health

## Abstract

**Introduction:**

Insomnia affects health, well-being, and productivity. Despite recommendations favoring Cognitive Behavioral Therapy for Insomnia (CBT-I) as first-line treatment, hypnotic medications remain widely used.

**Objective:**

To examine the frequency and types of sleep medications used by individuals with insomnia symptoms seeking psychological treatment, as well as factors associated with hypnotic use.

**Materials and Methods:**

The present cross-sectional study included 1,158 adults with insomnia symptoms recruited from a specialized service between 2021 and 2022. Participants completed online questionnaires assessing sociodemographic characteristics, medication use, and standardized measures of insomnia severity (Insomnia Severity Index), anxiety, and depression (Hospital Anxiety and Depression Scale). Descriptive analyses and multivariate logistic regression were performed.

**Results:**

From the total (n = 1,158), 60% of participants reported current use of sleep medication. The most frequently used classes were Z-drugs and benzodiazepines, followed by sedative antidepressants. Greater insomnia severity was the primary factor associated with hypnotic use (odds ratio = 1.14; *p* < 0.001). White participants were also more likely to use hypnotics, regardless of insomnia severity.

**Conclusions:**

Pharmacological treatment remains predominant among individuals with insomnia symptoms, despite recommendations for CBT-I as the first-line treatment. These findings highlight the importance of expanding access to behavioral interventions and promoting the rational use of sleep medications in Brazil.

## Introduction

Sleep disorders comprise a group of conditions characterized by disturbances in sleep quality, timing, and duration, with significant consequences for physical and mental health. Among these, insomnia is one of the most prevalent sleep disorders and is defined by persistent difficulties in initiating sleep, maintaining sleep, or experiencing early morning awakenings, accompanied by clinically significant daytime impairment. 1 These symptoms often occur in combination and reflect both nighttime disturbances and their broader functional consequences.

Epidemiological studies indicate that insomnia is highly prevalent, with approximately 30% of the general population reporting difficulties initiating or maintaining sleep, and about 10% meeting the Diagnostic and Statistical Manual of Mental Disorders, 5 ^th^ Edition's (DSM-5) criteria for insomnia disorder. 1 In Brazil, epidemiological data similarly indicate a high prevalence of sleep disturbances. A large national survey including more than 94,000 individuals reported that approximately 35% of the population experience sleep problems, with even higher rates observed in certain regions of the country. 2 Furthermore, studies focusing specifically on insomnia have found prevalence rates of approximately 15% based on diagnostic criteria, and up to 30% when assessed using objective measures. 3 These findings suggest that the burden of insomnia in Brazil is comparable to, or even exceeds, global estimates, and is associated with significant functional impairment and mental health burden in the population. 4

Insomnia often follows a chronic and recurrent course and is associated with substantial adverse effects on mental health, daily functioning, and socioeconomic outcomes. Moreover, it is linked to an increased risk of depression, anxiety, and cardiometabolic diseases, and is also recognized as a predictor of other mental disorders. 5 6

Cognitive and behavioral therapies for insomnia (CBT-I) are considered the first-line treatment, supported by robust evidence for their efficacy and long-term benefits. 7 8 Nonetheless, the prescription of hypnotic medications remains the most common management approach, particularly in primary care settings. 9 Benzodiazepines and nonbenzodiazepine hypnotics (Z-drugs)—including zolpidem, eszopiclone, and zaleplon—are among the most widely used pharmacological agents, often being prescribed beyond the recommended duration. 8

Although initially promoted as safer alternatives to benzodiazepines, accumulating evidence indicates that Z-drugs may be associated with serious adverse events, including tolerance, dependence, cognitive impairment, residual sedation, and increased risk of falls, particularly among older adults. 10 11 Additionally, observational studies have suggested potential associations between long-term use of hypnotics and increased risk of hospitalization and mortality. 10 Furthermore, when combined with opioids or benzodiazepines, these medications may increase the risk of fatal overdoses. 10 The off-label use of sedative antidepressants and antipsychotics for sleep has also been increasing. 12

However, in Brazil and other Latin American countries, data on real-world patterns of sleep medication use remain limited. Sociocultural and healthcare-related factors may influence patterns of sleep medication use in Latin American countries, particularly in Brazil. Epidemiological and pharmacoepidemiological data indicate relatively high rates of sleeping pill use and prolonged treatment duration in the Brazilian population. 13 These patterns may reflect the widespread use of pharmacological approaches in the management of sleep complaints in clinical practice. These contextual factors highlight the relevance of examining patterns of hypnotic use within the Brazilian setting.

While behavioral and cognitive interventions (e.g. CBT-I) are recognized as the preferred treatment for insomnia disorder, pharmacological therapy continues to dominate clinical practice worldwide. 8 14 15 Pharmacological treatments can be effective in the short term but demonstrate limited long-term efficacy and are associated with cumulative risks, including tolerance, dependence, cognitive impairment, and falls, particularly among older adults. 16 17 18 Despite international recommendations that hypnotics be prescribed for no longer than 4 weeks and ideally combined with behavioral interventions whenever possible, epidemiological studies continue to show persistently high rates of chronic prescriptions and long-term hypnotic use. 13 19 In Brazil, for instance, zolpidem consumption has increased by over 500% during the past decade, particularly among women and older adults. 13 20 This highlights a persistent gap between evidence-based guidelines and clinical practice.

The present study aimed to describe the frequency and types of sleep medications used by individuals with insomnia symptoms seeking non-pharmacological psychological treatment, as well as to identify sociodemographic and psychological factors associated with hypnotic use in this population. We hypothesized that there would be a high frequency of sleep medication use, particularly Z-drug hypnotics, among individuals seeking behavioral therapy for insomnia, and that medication use would be associated with greater severity of insomnia, anxiety, and depression symptoms.

## Materials and Methods

This cross-sectional study utilized data derived from research previously conducted by our research group. All data were obtained from research approved by the Ethics Committee of the University of São Paulo, and all participants provided informed consent prior to enrollment. Data management was performed using the Research Electronic Data Capture (REDCap, Vanderbilt University), a secure web platform designed for building and managing databases and online surveys.

### Participants and Procedure

Participants were individuals with insomnia symptoms enrolled in a randomized controlled trial evaluating the efficacy of Acceptance and Commitment Therapy for Insomnia (ACT-I) and CBT-I. 17 Detailed study procedures have been published previously. 21 Recruitment occurred between March 2021 and July 2022, through social media advertisements disseminated by the Institute of Psychiatry at the University of São Paulo. Interested volunteers accessed the REDCap platform and completed an online screening questionnaire to evaluate their eligibility based on predefined inclusion and exclusion criteria.

Eligibility criteria required that participants (a) be 18 to 59-years-old; and (b) meet the following criteria for chronic insomnia, according to DSM-5: (i) difficulty initiating and/or maintaining sleep (sleep-onset latency or wake after sleep onset ≥ 30 min); (ii) insomnia symptoms occurring > 3 nights per week for > 3 months; (iii) sleep disturbance (or associated daytime fatigue) that causes significant distress or impairment in social, occupational, or other functional areas; (iv) an Insomnia Severity Index (ISI) score ≥ 8. Participants who reported illiteracy or reading difficulties on the identification questionnaire were excluded.

Eligible participants received an email invitation to complete a series of instruments via the REDCap platform. The collected data included sociodemographic characteristics, information on sleep medication use, and standardized questionnaires assessing insomnia, depression, and anxiety. Participants did not receive any compensation for their participation.

### Measures

#### Participants' Data

Participants provided sociodemographic information, including age, sex, education level, marital status, and ethnicity. Two additional questions assessed physical activity: “Do you practice physical activity?” and “How often?”

#### Sleep Medication

Information on sleep medications was collected using the following questions: (1) “Do you use sleeping pills?” and (2) “If yes, how often?”. Continuous use was defined as sleep medication intake 5 or more nights per week, whereas occasional use was defined as fewer than 5 nights per week. This cut-off aligns with epidemiological studies that classify “most nights” or near-daily hypnotic use as 5 to 6 nights per week, 22 and was defined a priori to distinguish routine from intermittent patterns. Thus, medication use was operationalized based on the frequency of current weekly use (number of nights per week). To facilitate accurate reporting, participants were asked to indicate both the medication name and its pharmacological class. For each class, commonly used commercial names in Brazil were provided to support recognition and reduce recall errors.

#### Insomnia Severity Index (ISI)

The ISI, developed by Morin, 23 and validated in Portuguese by Castro, 24 is a retrospective seven-item scale assessing the nature, intensity, and impact of insomnia during the last month. Items evaluate difficulties with sleep onset, sleep maintenance, early morning awakening, degree of sleep satisfaction, daytime impairment, perceived sleep problems by others, and degree of sleep problem concern. Responses are rated on a five-point Likert scale (0 = no severity, to 4 = high severity), yielding a total score ranging from 0 to 28. Scores are categorized as follows: 0 to 7, no clinically significant insomnia; 8 to 14, mild; 15 to 21, moderate; and 22 to 28, severe insomnia. In the present sample, the ISI demonstrated high internal consistency (ω ≈ 0.95).

#### Hospital Anxiety and Depression Scale (HADS)

The HADS comprises 14 items divided into two subscales assessing anxiety (HADS-A) and depression (HADS-D). All items focus exclusively on emotional states, excluding somatic symptoms. Each subscale yields a total score ranging from 0 to 21, with scores of 0 to 8 indicating absence, and scores ≤ 9 indicating the presence of anxiety/depression. 25 The Brazilian version was translated and validated by Botega et al., 22 demonstrating Cronbach's alpha coefficients of 0.68 for anxiety and for depression 0.77. In the present sample, internal consistency was high for both the Anxiety (ω = 0.91) and Depression (ω = 0.88) subscales.

### Data Analysis

All statistical analyses were performed using R (Foundation for Statistical Computing), version 4.3.0. Statistical significance was set at *p* < 0.05 (two-tailed). Only participants with complete data for the relevant variables were included in each analysis. Descriptive statistics were computed to characterize the sample, with categorical variables expressed as frequencies and percentages, and continuous variables as means and standard deviations (SDs).

The sample was divided into two groups based on sleep medication use (“users” vs. “non-users”). Between-group comparisons were conducted using chi-squared tests for categorical variables and independent-sample *t* -tests for continuous variables.

To identify factors associated with sleep medication use, a multivariate logistic regression model was fitted, including the following predictors: age, sex, education, marital status, ethnicity, physical activity, insomnia severity (ISI score), anxiety (HADS-A), and depression (HADS-D). Because insomnia, anxiety, and depressive symptoms are partially correlated constructs, we evaluated potential multicollinearity among ISI, HADS-A and -D scores. Variance inflation factors (VIFs) were below commonly accepted thresholds (all < 3), indicating that collinearity was not problematic and that each construct contributed uniquely to the regression model.

Additionally, a Welch's *t* -test was conducted to examine whether the observed association between ethnicity and sleep medication use could be explained by differences in insomnia severity (ISI scores) across ethnic groups. Effect sizes were interpreted according to Cohen's guidelines, 26 where values of approximately 0.20 are considered small, 0.50 medium, and 0.80 large.

## Results

### Sample Characteristics

The study included 1,158 participants with a mean age of 38.9 years (SD = 10 years; range = 18–59 years). Most were female, held a university degree, and resided in the Southeast region of Brazil. Table 1 summarizes the means, SDs, and frequencies for sociodemographic characteristics, anxiety and depression scale scores, and physical activity practices in the overall sample.

**Table 1:** Descriptive analysis of the sample.

Variables	Non-users (n = 464) ^*a*^	Users (n = 694) ^*a*^	*p* -value *b*	Effect size c
**Age (years), mean (SD)**	38.79 (10.23)	39.24 (9.82)	0.5	
**Sex, n (%)**			0.034	0.05
Female	347 (75%)	555 (80%)		
Male	116 (25%)	137 (20%)		
**Physical activity, n (%)**			0.7	
≥ 2 times/week	241 (52%)	353 (51%)		
< 2 times/week	223 (48%)	340 (49%)		
**Education level, n (%)**			0.8	
Higher education	340 (74%)	511 (74%)		
Basic and secondary education	121 (26%)	177 (26%)		
**Marital status, n (%)**			0.3	
Married/Partnered	206 (44%)	330 (48%)		
Single/Divorced	258 (56%)	364 (52%)		
**Ethnicity, n (%)**			0.001	0.09
Black and Brown	159 (34%)	176 (26%)		
White	303 (66%)	511 (74%)		
**Region**			0.006	0.10
Federal District	6 (1.3%)	24 (3.5%)		
Central West	5 (1.1%)	25 (3.6%)		
Northeast	29 (6.3%)	59 (8.5%)		
North	11 (2.4%)	20 (2.9%)		
Southeast	373 (81%)	504 (73%)		
South	39 (8.4%)	62 (8.9%)		
**ISI**	17.71 (4.06)	19.93 (4.04)	< 0.001	-0.55
**Depression score (HADS-D)**	9.00 (4.22)	9.84 (4.42)	0.001	-0.19
**Anxiety score (HADS-A)**	10.92 (4.18)	12.00 (4.26)	< 0.001	-0.26

**Abbreviations:** HADS, hospital anxiety and depression scale; ISI, insomnia severity index; SD, standard deviation.

**Notes:**^a^ Mean (SD); n (%).

b One-way analysis of means (not assuming equal variances); Pearson's Chi-squared test.

c Cohen's d for continuous variables; Cramér's V for categorical variables.

### Patterns of Sleep Medication Use by Drug Type and Frequency

Table 2 summarizes the distribution of specific sleep medications among individuals categorized as occasional users (1–4 times per week) and continuous users (5–7 times per week). Zolpidem was the most frequently reported medication in both groups, with a significantly higher prevalence among continuous users ( *p* < 0.001). Other medications more commonly used on a continuous basis included trazodone, mirtazapine, pregabalin, and quetiapine. In contrast, the use of muscle relaxants and antihistamines was more prevalent among occasional users. These patterns suggest that continuous users tend to rely more on pharmacological agents typically prescribed for chronic insomnia or comorbid conditions, whereas occasional users more often report medications associated with transient or situational sleep difficulties.

**Table 2:** Sleep medication use by drug type: occasional vs. continuous.

Medication name	Class/Pharmacological group	Occasional use (n = 252)	Continuous use (n = 442)	*p* -value
Alprazolam	Benzodiazepine (anxiolytic/hypnotic)	24 (9.5%)	59 (13%)	0.14
Bromazepam	Benzodiazepine (anxiolytic)	6 (2.4%)	8 (1.8%)	0.6
Clonazepam	Benzodiazepine (anxiolytic/anticonvulsant)	50 (20%)	102 (23%)	0.3
Diazepam	Benzodiazepine (anxiolytic/muscle relaxant)	5 (2.0%)	9 (2.0%)	> 0.9
Zolpidem	Non-benzodiazepine hypnotic (Z-drug)	82 (33%)	202 (46%)	< 0.001
Eszopiclone	Non-benzodiazepine hypnotic (Z-drug)	6 (2.4%)	21 (4.8%)	0.12
Amitriptyline	Tricyclic antidepressant (sedative properties)	8 (3.2%)	29 (6.6%)	0.056
Doxepin	Tricyclic antidepressant (sedating, low dose used for insomnia)	2 (0.8%)	3 (0.7%)	>0.9
Trazodone	SARI (sedative)	24 (9.5%)	80 (18%)	0.002
Mirtazapine	NaSSA (sedative)	2 (0.8%)	23 (5.2%)	0.003
Gabapentin	Anticonvulsant/Neuromodulator (off-label for sleep)	0 (0%)	6 (1.4%)	0.092
Pregabalin	Anticonvulsant/ Neuromodulator (off-label for sleep/anxiety)	5 (2.0%)	47 (11%)	< 0.001
Olanzapine	Atypical antipsychotic (sedative properties)	0 (0%)	3 (0.7%)	0.6
Quetiapine	Atypical antipsychotic (sedative, low-dose use common for sleep)	22 (8.7%)	75 (17%)	0.003
Ramelteon	MT1/MT2	0 (0%)	5 (1.1%)	0.2
Agomelatine	Melatonergic antidepressant (MT1/MT2 agonist, 5-HT2C antagonist)	3 (1.2%)	3 (0.7%)	0.7
Antihistamines	H1 receptor antagonists (sedative)	24 (9.5%)	24 (5.4%)	0.041
Muscle relaxants	Skeletal muscle relaxants (various mechanisms)	94 (37%)	61 (14%)	< 0.001
Dimenhydrinate	Antihistamine/Antiemetic (sedative effects)	44 (17%)	27 (6.1%)	< 0.001

**Abbreviations:** 5-HT2C, 5-hydroxytryptamine, 5-HT; MT, melatonin receptor agonist; NaSSA, noradrenergic and specific serotonergic antidepressant; SARI, serotonin antagonist and reuptake inhibitor.

Regarding prescription status, most participants who reported using sleep medication indicated that their medication had been prescribed by a healthcare professional (n = 420; 60%), whereas 274 (40%) reported using sleep aids without medical prescription. Among nonprescription users, the most frequently reported drugs were over-the-counter antihistamines and muscle relaxants, while prescribed users more commonly reported Z-drugs and benzodiazepines. The results highlight that, although prescription-based use predominated, a non-negligible proportion of participants relied on nonprescribed sleep medications. No significant differences were found in insomnia severity between prescribed and nonprescribed users ( *p* > 0.05).

**Fig. 1Note:**
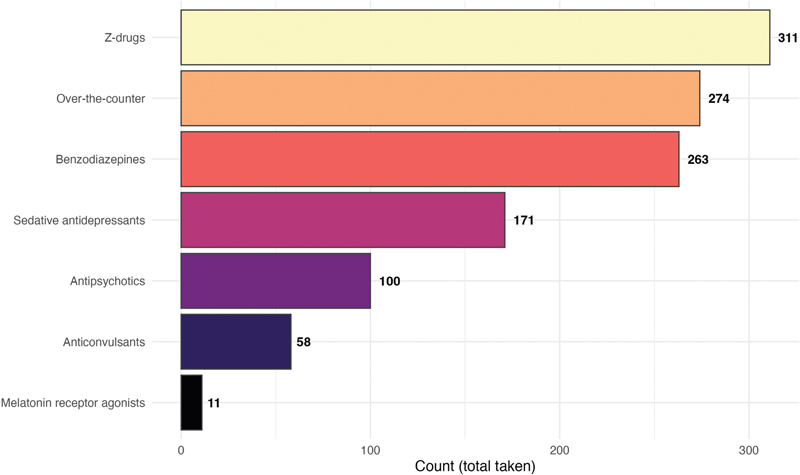
Distribution of reported sleep medications in the total sample, including pharmacological classes and over-the-counter medications. : Medication classes were not mutually exclusive; participants could report the use of more than one sleep medication. “Over-the-counter” refers to non-prescription medications rather than a pharmacological class.

Fig. 1 shows the distribution of the most frequently used classes of sleep medications in the total sample. Nonbenzodiazepine hypnotics (Z-drugs) were the most commonly reported category ( *n* = 311), followed by over-the-counter medications ( *n* = 274), benzodiazepines ( *n* = 263), sedative antidepressants ( *n* = 171), antipsychotics ( *n* = 100), anticonvulsants ( *n* = 58), and melatonin receptor agonists ( *n* = 11). Medication categories were not mutually exclusive, and participants could report the use of more than one sleep medication.

### Factors Associated with Sleep Medication Use

Table 3 presents the results of the logistic regression analysis examining sociodemographic and psychological factors associated with sleep medication use. The model demonstrated modest explanatory power (Tjur's R ^2^ = 0.08; Akaike Information Criterion [AIC] = 1,359.9; Bayesian Information Criterion [BIC] = 1,409.6). Higher insomnia severity (ISI score) was significantly associated with greater odds of medication use (odds ratio [OR] = 1.14; 95% confidence interval [CI]: 1.10, 1.18; *p* < 0.001). Participants who self-identified as White also had higher odds of using sleep medication compared to Black and Brown participants (OR = 1.55; 95% CI: 1.17, 2.06; *p* < 0.001). No significant associations were observed for age, sex, educational level, marital status, physical activity, or anxiety and depression scores.

**Table 3:** Factors associated with sleep medication use.

Variables	OR [95% CI]
(Intercept)	0.09** [0.04, 0.22]
Age	1.00 [0.99, 1.02]
Sex (male)	0.75 [0.55, 1.02]
Education level (basic and secondary)	0.94 [0.69, 1.28]
Marital status (single/divorced)	0.90 [0.69, 1.18]
Ethnicity (White)	1.55** [1.17, 2.06]
Physical activity, (< 2 times/week)	0.92 [0.70, 1.20]
Insomnia score	1.14** [1.10, 1.18]
Anxiety score	1.02 [0.98, 1.06]
Depression score	1.00 [0.96, 1.04]
Number of observations	1,067
AIC	1,359.9
BIC	1,409.6
Log.Lik.	–669.939
RMSE	0.47

**Abbreviations:** AIC, Akaike information criterion; BIC, Bayesian information criterion; CI, confidence interval; OR, odds ratio; RMSE, root mean square error.

**Notes:** * *p* < 0.05. ** *p* < 0.001.

Overall, these findings suggest that insomnia severity is the primary factor associated with sleep medication use, whereas sociodemographic and emotional variables play a comparatively minor role in explaining this behavior within the sample.

To explore whether the association between ethnicity and sleep medication use observed in the logistic regression could be attributed to differences in insomnia severity, a Welch's *t* -test was conducted to compare ISI scores between participants self-identifying as Black or Brown and those identifying as White. The results indicated no significant difference in ISI scores between the two groups ( *M* = 19.21 for Black/Brown vs. 19.02 for White; *t* (576.25) = 0.69; *p* = 0.493). The effect size was small (Cohen's *d* = 0.05; 95% CI: -0.09, 0.18), suggesting that insomnia severity did not differ meaningfully by ethnicity. Therefore, the higher likelihood of sleep medication use among White participants cannot be attributed to differences in severity.

## Discussion

The findings of this study suggest that sleep medication use is highly prevalent among individuals with insomnia symptoms, including those seeking nonpharmacological psychological interventions. The most frequently used classes were Z-drugs and benzodiazepines, followed by sedatives, antidepressants, and off-label antipsychotics. Although medications such as trazodone, quetiapine, and mirtazapine may be prescribed for a range of psychiatric or medical conditions, they are commonly used as sleep aids, which justifies their inclusion in the category of sleep medications in this study. These results mirror international trends, which identify zolpidem and trazodone as some of the most prescribed agents for insomnia. 14 27 Despite international guidelines recommending short-term use of hypnotics, 5 the high rate of continuous use observed in our sample highlights a persistent gap between clinical practice and evidence-based recommendations.

Insomnia severity was the factor most strongly associated with sleep medication use, consistent with previous studies showing that more severe symptoms predict a greater likelihood of pharmacological treatment. 7 28 In our sample, White participants were more likely to use sleep medication despite comparable insomnia severity across ethnic groups. This pattern may be related to sociocultural or access-related disparities in prescription practices and the availability of behavioral sleep therapies, as well as broader structural inequalities in healthcare access, which may influence both the likelihood of receiving pharmacological treatment and patterns of healthcare utilization. While population-based studies, such as Bertisch et al., 27 have described clinical and demographic correlates of hypnotic use in the United States, further research is needed to clarify the role of ethnicity and healthcare access in sleep medication use within Latin American contexts.

Additionally, a substantial proportion of participants reported using sleep medications without a medical prescription, mostly over-the-counter antihistamines and muscle relaxants. This finding suggests that self-medication practices may play a role in the widespread use of sleep aids in Brazil, potentially reflecting structural barriers to accessing specialized sleep care, as well as broader inequalities in healthcare access. These factors may contribute to the use of readily available medications, particularly among individuals facing greater constraints in accessing formal health services.

Beyond nonprescribed use, previous have also documented a rapid growth in the dispensing of prescription hypnotics, particularly zolpidem, underscoring the regulatory challenges in monitoring and controlling hypnotic sales and long-term use. 13 20 Public health initiatives and tighter control over both nonprescribed and prescribed medication may be important to mitigate risks associated with misuse and dependence.

The regression model showed limited explanatory power, suggesting that factors beyond individual symptoms and demographic variables may influence hypnotic use in this population. Other clinical and contextual factors not assessed in this study—such as chronic pain, substance use, comorbid medical conditions, and type of healthcare services accessed (e.g., public vs. private)—may also contribute to hypnotic use and should be examined in future research. Neither anxiety nor depression symptoms were independently associated with medication use after controlling for insomnia severity, suggesting that hypnotic use may be more closely related to sleep-related distress than to general emotional distress. The higher likelihood of hypnotic use among White participants, even after adjusting for insomnia severity, may reflect structural and cultural factors—such as differences in mental health access, prescribing practices, and attitudes toward medication use, which warrant further investigation in the Brazilian context.

Recent studies have raised concerns about the prolonged and potentially unsafe use of hypnotics, particularly Z-drugs, due to risks of tolerance, dependence, falls, cognitive impairment, and fatal overdoses when combined with opioids or benzodiazepines. 10 Conversely, systematic reviews and meta-analyses have demonstrated that psychological interventions, such as CBT-I and ACT-I, produce more durable and sustained improvements in daytime functioning and carry fewer risks. 7 12 These findings are consistent with the importance of promoting rational hypnotic use and expanding access to evidence-based behavioral interventions.

Furthermore, the present study's results align with international evidence showing persistently high rates of hypnotic use, despite recommendations for short-term treatment. Systematic reviews and meta-analyses indicate that, although drugs such as eszopiclone and zolpidem are effective for acute insomnia, their prolonged use is associated with increased risks of adverse effects and negative health outcomes. 14 15 18 In Brazil, zolpidem remains the most widely used hypnotic, with prescriptions increasing significantly over the past decade, reflecting a growing reliance on pharmacological approaches. 13 20 These findings underscore the need for supervised deprescribing strategies and expanded access to behavioral therapies, which offer safer and more effective long-term outcomes. 15 19

This study has several limitations. First, as in most online epidemiological surveys, our sample was subject to selection bias. Participants were recruited through digital platforms and a sleep-focused research service, which likely resulted in an overrepresentation of individuals who are more educated, digitally literate, and motivated to seek psychological treatment. Although this sampling strategy limits the generalizability of the findings to the broader Brazilian population, it does not compromise the internal validity of the associations investigated. The study's aim was not population estimation but rather the characterization of medication use patterns among adults seeking help for insomnia symptoms in real-world settings.

Second, medication use and clinical symptoms were based on self-report, which introduces the possibility of information bias, including recall bias, with potential inaccuracies in reporting medication names, dosages, and duration of use. To mitigate this limitation, structured questions were used to assess current use and frequency, and participants were asked to report both the medication name and its pharmacological class. Additionally, commonly used commercial names in Brazil were provided to facilitate recognition and reduce recall errors. Nevertheless, chronic users may report medication details more accurately than occasional users, which may lead to differential misclassification. This limitation is inherent to large-scale online surveys and should be interpreted accordingly. Despite this, the instruments employed are widely validated self-report measures, and the patterns observed are consistent with prior literature on hypnotic use.

Third, the sample was not representative of the general Brazilian population, as individuals seeking nonpharmacological psychological treatment for insomnia were recruited through a research protocol conducted in a specialized university-based sleep service. Participants tended to be highly educated and predominantly White, which limits the generalizability of the findings.

Fourth, the cross-sectional design precludes causal inferences, as the temporal direction of the associations cannot be established. Fifth, sleep medication use was assessed through self-report, without verification of prescriptions, doses, or duration of treatment. Although participants indicated whether their medication had been prescribed by a healthcare professional, it was not possible to determine the medical specialty involved or confirm potential cases of self-medication. This limitation prevented a more detailed analysis of prescribing patterns and potential differences between professional categories (e.g., general practitioners, psychiatrists, or sleep medicine specialists). Additionally, the duration of medication use (e.g., cumulative months or long-term use) was not assessed in this study.

Despite these limitations, the findings emphasize the need for further research using larger, more diverse, and nationally representative samples to examine regional, cultural, and healthcare access differences in sleep medication use across Brazil.

The present findings have important clinical implications. The high frequency of hypnotic use observed in this sample highlights the need for systematic assessment of medication use among individuals presenting with insomnia symptoms, particularly in settings where patients seek nonpharmacological treatment. Clinicians should carefully evaluate the indication, duration, and ongoing necessity of hypnotic prescriptions, with particular attention to long-term use. These findings also underscore the importance of screening for potential adverse effects and monitoring patterns of use over time. Additionally, the results reinforce the need to promote and expand access to evidence-based, nonpharmacological interventions, such as cognitive behavioral therapy for insomnia, as first-line treatment options.

Overall, these findings highlight the relevance of clinical and public health strategies that integrate psychotherapy with responsible pharmacological management, including supervised deprescribing programs and broader implementation of brief behavioral treatments for insomnia. Incorporating such approaches in primary care and specialized settings may contribute to reducing inappropriate hypnotic use and improve the quality of insomnia care in Brazil.

## Conclusion

Sleep medication use is frequent among adults with insomnia symptoms, but population-level data in Brazil remain scarce. Prior studies suggested high rates of hypnotic use and limited access to behavioral treatments, yet little was known about real-world medication patterns among individuals actively seeking nonpharmacological care.

This study provides the first large-scale characterization of sleep medication use among Brazilian adults with insomnia symptoms. It identifies the most frequently used drug classes, patterns of continuous versus occasional use, as well as sociodemographic and clinical factors associated with hypnotic use, offering data relevant for clinical practice and public health policy.
